# Comparative immunogenicity of COVID-19 vaccination in pregnant and non-pregnant women: a real-world cohort study

**DOI:** 10.3389/fpubh.2026.1778064

**Published:** 2026-05-15

**Authors:** Altan Aksoy, Ayşegül Karahanoğlu, Salih Cesur, Rukiye Berkem, Ayşe Esra Karakoç, Yusuf Üstün, Sami Kınıklı, Mehmet Emin Demir

**Affiliations:** 1 Department of Medical Microbiology, Ankara Training and Research Hospital, Ankara, Türkiye; 2 Clinic of Obstetrics and Gynecology, Ankara Training and Research Hospital, Ankara, Türkiye; 3 Clinic of Infectious Diseases and Clinical Microbiology, Ankara Training and Research Hospital, Ankara, Türkiye; 4 School of Medicine, Medicana International Ankara Hospital, Atılım University, Ankara, Türkiye

**Keywords:** booster vaccination, COVID-19, humoral immunity, neutralizing antibodies, pregnancy

## Abstract

**Background:**

Pregnant individuals are at increased risk of severe COVID-19-related morbidity and adverse obstetric outcomes, highlighting the importance of effective vaccination. Although COVID-19 vaccine safety during pregnancy is well established, real-world evidence on vaccine-induced humoral immunity remains limited. This study compared neutralizing antibody responses after COVID-19 vaccination in pregnant and non-pregnant women and evaluated the effects of vaccine dose number, platform, and timing during pregnancy.

**Methods:**

This retrospective cohort study included 171 vaccinated women of reproductive age, comprising 95 pregnant and 76 non-pregnant participants. Neutralizing antibody titers were measured using a surrogate virus neutralization test. Vaccine platforms included mRNA vaccines (BNT162b2, Pfizer–BioNTech), inactivated vaccines (CoronaVac, Sinovac), and a small number of heterologous regimens. Antibody levels were compared using the Mann–Whitney *U* test. Associations between antibody titers and vaccine dose number, age, gestational age, and trimester were assessed using Spearman correlation analyses and Jonckheere–Terpstra trend tests.

**Results:**

Pregnant women had significantly lower neutralizing antibody titers than non-pregnant controls (median 5.62 vs. 16.25 AU/mL; *p* = 0.008) and were less likely to reach the assay’s upper detection limit (≥30 AU/mL: 18.9% vs. 43.4%). Although the categorical distribution of vaccine dose counts was broadly similar between groups (*χ*^2^*p* = 0.098), the mean number of doses was significantly lower among pregnant women (1.93 ± 0.72 vs. 2.32 ± 1.02; *p* = 0.011), reflecting differences in cumulative vaccine exposure at higher dose levels. Antibody titers were strongly associated with vaccine dose number overall (*ρ* = 0.341, *p* < 0.001) and in both pregnant and non-pregnant women. When stratified by dose category, antibody titers did not differ by pregnancy status. Gestational age and trimester were not independently associated with antibody levels, although the dose–response relationship was strongest in second-trimester vaccination. In unadjusted comparisons, mRNA vaccines were associated with numerically higher titers than inactivated vaccines (median 11.30 vs. 7.72 AU/mL; *p* = 0.165). mRNA platform was also independently associated with higher titers in multivariable analysis (*β* = 0.721, *p* = 0.030); this finding should be interpreted cautiously given the small inactivated-vaccine subgroup (*n* = 18).

**Conclusion:**

COVID-19 vaccination elicits robust neutralizing antibody responses in pregnant women, with overall humoral responsiveness preserved despite quantitative differences in antibody magnitude. Lower antibody levels are predominantly explained by differences in cumulative vaccine exposure rather than pregnancy-related immune impairment, although a residual independent association of pregnancy cannot be fully excluded. Completing recommended vaccine schedules is essential to optimize maternal and neonatal protection.

## Introduction

COVID-19 poses heightened risks in pregnancy. Pregnant individuals with symptomatic SARS-CoV-2 infection have significantly higher rates of intensive care unit admission, mechanical ventilation, and mortality compared to non-pregnant women of reproductive age (1–3). Infection during pregnancy is also associated with adverse outcomes such as preterm birth and stillbirth (4–6). These risks underscore the importance of effective vaccination in this population. Early in the pandemic, however, pregnant women were excluded from vaccine trials, leading to initial hesitancy and low vaccine uptake among pregnant people (7). Despite concerns, extensive real-world data now confirm that COVID-19 vaccines are safe in pregnancy and not associated with adverse obstetric outcomes (7, 8). No increase in miscarriage, congenital anomalies, or stillbirth has been observed following maternal vaccination (9). On the contrary, some studies even suggest vaccination during pregnancy may reduce the risk of stillbirth and other complications (10, 11).

Pregnancy involves immune adaptations, raising questions about how it may affect vaccine-induced immunity. Early studies showed that pregnant and lactating women mounted humoral and cellular responses to mRNA COVID-19 vaccines comparable to non-pregnant women, with IgG antibodies effectively crossing the placenta and appearing in breastmilk (1, 6, 12). Two doses were necessary to achieve similar antibody levels, and booster doses during pregnancy further elevated titers, including against variants like Omicron (13). Mechanistically, T-cell and memory B-cell responses remain largely intact in pregnancy, though minor differences (such as altered Fc profiles and slightly narrower neutralization breadth) have been observed (14).

Pregnant individuals often receive fewer COVID-19 vaccine doses or delay vaccination due to safety concerns, which may result in lower antibody levels despite intact immune capacity. We hypothesized that differences in cumulative vaccine exposure—rather than intrinsic immune impairment—primarily account for lower antibody levels observed in pregnant women. To investigate this, we compared humoral immunogenicity between vaccinated pregnant and non-pregnant women, focusing on neutralizing antibody titers as a key correlate of protection. We analyzed associations with vaccine dose number, type, and timing (trimester), while also accounting for potential confounders such as prior infection and time since vaccination.

## Methods

### Study design and participants

We conducted a retrospective cohort study including women of reproductive age who had received COVID-19 vaccination, comparing pregnant women with non-pregnant controls. Participants were recruited from a single tertiary-care academic hospital and its affiliated outpatient clinics. The study period spanned 2021-2022, during which both mRNA-based and inactivated SARS-CoV-2 vaccines were administered as part of the national vaccination program.

Eligible participants were women aged 18–45 years with documented receipt of at least one dose of a COVID-19 vaccine. The pregnant cohort comprised women vaccinated during an ongoing singleton pregnancy at any gestational week across all three trimesters. The non-pregnant control group consisted of women vaccinated during the same time frame who were not pregnant at vaccination or blood sampling. Women with multifetal pregnancies, known immunodeficiency, chronic immunosuppressive therapy, or incomplete vaccination records were excluded. The study was approved by the Institutional Ethics Committee of Ankara Training and Research Hospital (17.04.2024; IRB: E-24-62), and written informed consent was obtained from all participants.

### Vaccination details

Vaccines administered included mRNA-based vaccines (BNT162b2, Pfizer–BioNTech; and, in a small number of cases, mRNA-1273, Moderna) and an inactivated whole-virus vaccine (CoronaVac, Sinovac). Vaccine type, manufacturer, total number of doses received, and dates of administration were recorded for each participant. Participants were categorized according to vaccine platform (mRNA versus inactivated) and total vaccine dose number. A small subset received heterologous vaccination schedules (mixed mRNA and inactivated vaccines); these individuals were analyzed descriptively and excluded from direct platform-specific comparisons to avoid misclassification bias.

### Data and specimen collection

Demographic and clinical data were obtained from electronic medical records and standardized questionnaires, including age, pregnancy status, gestational age at vaccination and blood sampling, vaccine platform, number of doses received, and interval between the last vaccine dose and blood collection. Information on prior SARS-CoV-2 infection was obtained from self-report and available clinical records.

Venous blood samples were collected approximately 4–12 weeks after the most recent vaccine dose to allow for comparable antibody maturation and early waning intervals. For pregnant participants, gestational age at blood draw was recorded. All samples were processed under standardized laboratory conditions and stored at −80 °C until analysis.

### Neutralizing antibody assay

Neutralizing antibody levels against SARS-CoV-2 were quantified using a surrogate virus neutralization test (sVNT; GenScript cPass SARS-CoV-2 Neutralization Antibody Detection Kit, Piscataway, NJ, United States). This competitive ELISA-based assay measures antibodies that inhibit the interaction between the viral receptor-binding domain (RBD) and the human ACE2 receptor, serving as a validated surrogate for neutralization capacity. All assays were performed according to the manufacturer’s protocol at a single serum dilution of 1:10. Higher serial dilutions were not performed due to assay protocol constraints and real-world laboratory workflow requirements; this limited the upper dynamic range but ensured methodological consistency across all samples. Results were expressed in arbitrary units per milliliter (AU/mL), with a manufacturer-recommended positivity threshold corresponding to 30% inhibition. Semi-quantitative AU/mL values were derived from assay-specific calibration curves provided by the manufacturer’s analysis software rather than direct reporting of percent inhibition values. A formal conversion to WHO International Units (IU/mL) using the NIBSC 20/136 international standard was not performed; this limits direct comparability with studies reporting titers in IU/mL. All participants demonstrated detectable neutralizing activity. Due to the assay’s upper detection limit, values ≥30 AU/mL were recorded as 30 AU/mL in nonparametric analyses. A subset of samples was additionally evaluated using a live-virus plaque reduction neutralization test (PRNT_50_) for validation, demonstrating a strong rank correlation with sVNT results (Spearman *ρ* = 0.82, *p* < 0.001).

### Outcomes

The primary outcome was the serum neutralizing antibody titer measured by sVNT. Secondary outcomes included the relationship between antibody titers and vaccine dose number, vaccine platform, and timing of vaccination during pregnancy. Breakthrough SARS-CoV-2 infections occurring more than 14 days after the final vaccine dose were recorded descriptively.

### Sample size considerations and reporting

The sample size was determined by the number of eligible participants during the study period (95 pregnant and 76 non-pregnant women). *Post hoc* power analysis indicated adequate power to detect moderate differences in antibody titers. All primary analyses were prespecified; additional multivariable and sensitivity analyses were performed to further assess robustness. The study was conducted and reported in accordance with the STROBE guidelines.

### Statistical analysis

Statistical analyses were performed using IBM SPSS Statistics (version 22.0; IBM Corp., Armonk, NY, United States). Continuous variables were summarized as median with range or interquartile range (IQR) for non-normally distributed data and as mean ± standard deviation (SD) when appropriate. Categorical variables were reported as counts and percentages. Normality of neutralizing antibody titers was assessed using the Shapiro–Wilk test and visual inspection; as titers were right-skewed, nonparametric methods were applied. Neutralizing antibody levels were compared between pregnant and non-pregnant women using the Mann–Whitney *U* test. Baseline characteristics were compared using the Mann–Whitney *U* test for continuous variables and Pearson’s chi-square test for categorical variables. Associations between antibody titers and vaccine dose number, age, gestational age, and time since last vaccination were evaluated using Spearman’s rank correlation coefficients, with analyses performed in the overall cohort and stratified by pregnancy status. Comparisons across multiple groups were conducted using the Kruskal–Wallis test with Dunn’s *post hoc* analysis. Dose–response trends across ordered groups were assessed using the Jonckheere–Terpstra trend test. To formally assess independent predictors of antibody response, two multivariable models were constructed on participants with complete covariate data (*n* = 163; 8 participants with heterologous vaccine regimens excluded). The first was a multivariable linear regression with log-transformed antibody titers [ln (titer + 0.01)] as the outcome, adjusting simultaneously for pregnancy status, vaccine dose count, age, prior SARS-CoV-2 infection, and vaccine platform. Log-transformation was chosen to address the right-skewed distribution and the impact of right-censoring at the upper detection limit. The interval since last vaccination was evaluated as a candidate covariate but was excluded from the final model due to lack of independent association (Spearman *ρ* = −0.15, *p* = 0.06; model *p* > 0.10), minimizing the risk of overfitting. The second was a multivariable logistic regression using a binary outcome (reaching ≥30 AU/mL) to directly address the ceiling effect. Variance inflation factors (VIF) were calculated for all predictors to formally assess multicollinearity. Missing data were minimal and were not imputed. All tests were two-tailed with a significance threshold of *p* < 0.05.

## Results

The study included 171 women: 95 pregnant and 76 non-pregnant controls. As detailed in Table 1, pregnant participants were significantly younger, with a median age of 27.0 years (range 18–40) compared to 29.5 years (range 19–43) in non-pregnant women (*p* = 0.047). All participants had received at least one COVID-19 vaccine dose, and approximately 80% of the cohort (136/171) had received at least two vaccine doses.

**Table 1:** Clinical characteristics of the pregnant and control groups.

Characteristic	Control (*n* = 76)	Pregnant (*n* = 95)	*p*-value
Age (years), median [range]	29.5 [19–43]	27.0 [18–40]	0.047^a^
Vaccine type, *n* (%)			0.163^b^
BNT162b2 (BioNTech)	60 (78.9%)	85 (89.5%)	
CoronaVac (Sinovac)	11 (14.5%)	7 (7.4%)	
Mixed (BNT162b2 + CoronaVac)	5 (6.6%)	3 (3.2%)	
Vaccine dose count, *n* (%)			0.098^b^
1 dose	10 (13.2%)	25 (26.3%)	
2 doses	46 (60.5%)	55 (57.9%)	
3 doses	11 (14.5%)	12 (12.6%)	
≥4 doses	9 (11.8%)	3 (3.2%)	
Vaccine doses (continuous): mean ± SD	2.32 ± 1.02	1.93 ± 0.72	0.011^a^
Ordinal trend (linear-by-linear)			0.004^b^
Neutralizing antibody (AU/mL), median [range]	16.25 [0.3–30.0]	5.62 [0.2–30.0]	0.008^a^
Prior SARS-CoV-2 infection, *n* (%)	25 (32.9%)	22 (23.2%)	0.256^b^
Time since last vaccine dose (weeks)^c^	7.5 (5.2–9.8)	8.1 (6.0–10.2)	0.345

a Mann–Whitney *U* test.

b Pearson Chi-square test (*χ*^2^).

c Blood sampling was scheduled within a predefined uniform 4–12 week post-vaccination window for all participants.

The interval from the last vaccine dose to sampling showed only a weak and non-significant inverse correlation with antibody levels (*ρ* = −0.15, *p* = 0.06). Importantly, the actual time since the last vaccine dose did not differ significantly between non-pregnant (median 7.5 weeks, IQR 5.2–9.8) and pregnant participants (median 8.1 weeks, IQR 6.0–10.2; Mann–Whitney *U* test, *p* = 0.345), confirming that differential waning is unlikely to have biased the group comparisons.

Vaccine type distribution was broadly similar between groups (*p* = 0.163). The majority in both cohorts received mRNA vaccines (89.5% of pregnant and 78.9% of non-pregnant participants). A smaller proportion received only an inactivated vaccine (7.4% vs. 14.5%, respectively), and very few received mixed-platform schedules (3.2% vs. 6.6%, respectively). The categorical distribution of vaccine dose counts was broadly similar between groups (1 dose: 13.2% vs. 26.3%; 2 doses: 60.5% vs. 57.9%; 3 doses: 14.5% vs. 12.6%; 4 + doses: 11.8% vs. 3.2%; *χ*^2^ *p* = 0.098). However, the mean number of doses was significantly lower in pregnant women (1.93 ± 0.72 vs. 2.32 ± 1.02; *p* = 0.011), and a significant ordinal trend toward fewer doses in the pregnant cohort was observed (linear-by-linear association, *p* = 0.004), reflecting differences in cumulative vaccine exposure at higher dose levels (Table 1). Prior SARS-CoV-2 infection rates did not differ significantly between groups (non-pregnant: 32.9%; pregnant: 23.2%; *p* = 0.256), confirming baseline balance for this potential confounder (Table 1).

**Figure 1:**
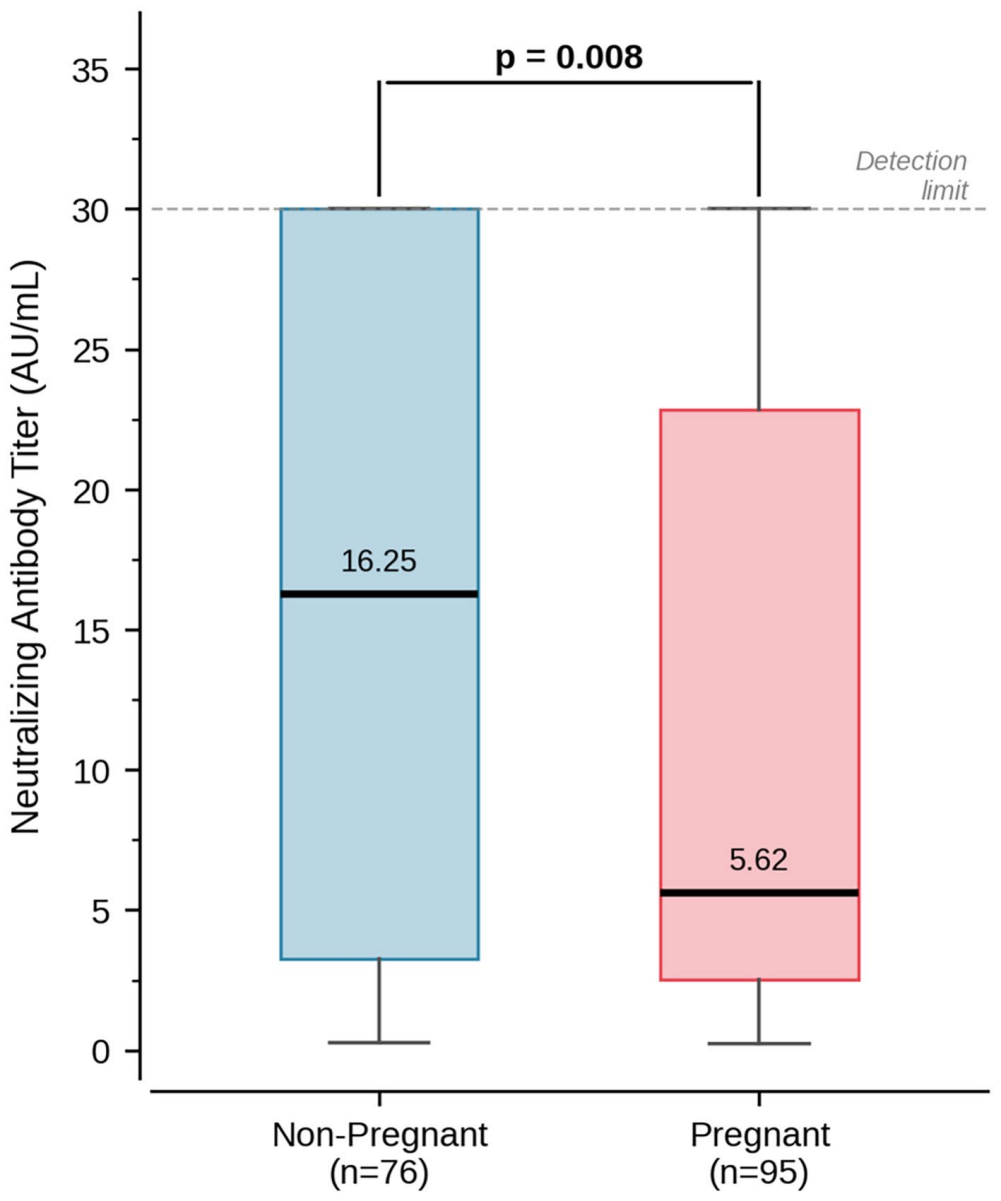
Distribution of SARS-CoV-2 neutralizing antibody titers in pregnant and non-pregnant women. Box plots display the median (horizontal line), interquartile range (box), and range (whiskers). Pregnant women had significantly lower antibody titers compared to non-pregnant controls (median 5.62 vs. 16.25 AU/mL; Mann–Whitney *U* test, *p* = 0.008).

Pregnant women had significantly lower SARS-CoV-2 neutralizing antibody titers than non-pregnant women (median 5.62 vs. 16.25 AU/mL; *p* = 0.008) (Table 1, Figure 1). Consistent with this, a smaller proportion of pregnant women reached the assay’s upper limit of detection (≥30 AU/mL: 18.9% vs. 43.4%), while low titers (<1 AU/mL) were more common (9.5% vs. 3.9%). These findings indicate a generally lower magnitude of humoral response in pregnant women, a difference attributable to differences in cumulative vaccine exposure rather than impaired immune responsiveness.

When stratified by vaccine dose count, no statistically significant differences in neutralizing antibody titers were observed between pregnant and non-pregnant women within any individual dose category (Table 2). Among participants with two doses (*n* = 101), antibody levels were numerically lower in pregnant women, although the difference did not reach statistical significance (median 5.22 vs. 13.30 AU/mL; *p* = 0.070). A similar non-significant trend was observed among those receiving three doses (median 27.25 vs. 30.00 AU/mL; *p* = 0.120). The ordinal distribution of vaccine doses showed a significant trend toward fewer doses in the pregnant cohort (linear-by-linear association, *p* = 0.004), with pregnant women underrepresented in the ≥4-dose category (3.2% vs. 11.8%) (Table 2).

**Table 2:** Neutralizing antibody titers by vaccine dose count.

Dose	NP (*n*)	NP titer, median (IQR)	Preg (*n*)	Preg titer, median (IQR)	*p* ^a^
1 dose	10	4.48 (1.84–14.55)	25	3.09 (1.18–13.90)	0.701
2 doses	46	13.30 (3.33–30.00)	55	5.22 (2.96–18.35)	0.070
3 doses	11	30.00 (30.00–30.00)	12	27.25 (3.50–30.00)	0.120
≥4 doses	9	30.00 (3.23–30.00)	3	30.00 (17.71–30.00)	0.680
≥3 doses (combined)^b^	20	30.00 (26.78–30.00)	15	30.00 (4.51–30.00)	0.431

a Mann–Whitney *U* test. NP, non-pregnant; Preg, Pregnant; IQR, interquartile range.

b The ≥3 doses combined row is provided to facilitate comparison with Figure 2, which displays 1-dose, 2-dose, and ≥3-dose categories. Combining the 3-dose stratum (NP *n* = 11; Preg *n* = 12) and ≥4-dose stratum (NP *n* = 9; Preg *n* = 3) yields *n* = 20 (NP) and *n* = 15 (Pregnant). Both groups show identical median of 30.00 AU/mL (assay ceiling), consistent with the individual strata. This combined category is displayed in Figure 2 to avoid sparse cells; individual strata are shown above for full transparency.

The combined ≥3-dose NP IQR (Q1 = 26.77 AU/mL) is higher than the ≥4-dose NP IQR (Q1 = 3.23 AU/mL). This apparent paradox reflects a well-known composition effect: the 3-dose NP subgroup is heavily right-censored (10/11 values at assay ceiling; Q1 = 30.00 AU/mL), which elevates the combined Q1 when both strata are merged. Both IQR values are mathematically verified from the raw data.

In the combined cohort, neutralizing antibody titers demonstrated a significant positive association with the number of vaccine doses received (Spearman’s *ρ* = 0.341, *p* < 0.001) (Figure 2). This dose–antibody relationship remained consistent when analyses were stratified by pregnancy status, with significant correlations observed in both non-pregnant women (*ρ* = 0.306, *p* = 0.007) and pregnant women (*ρ* = 0.310, *p* = 0.002).

**Figure 2:**
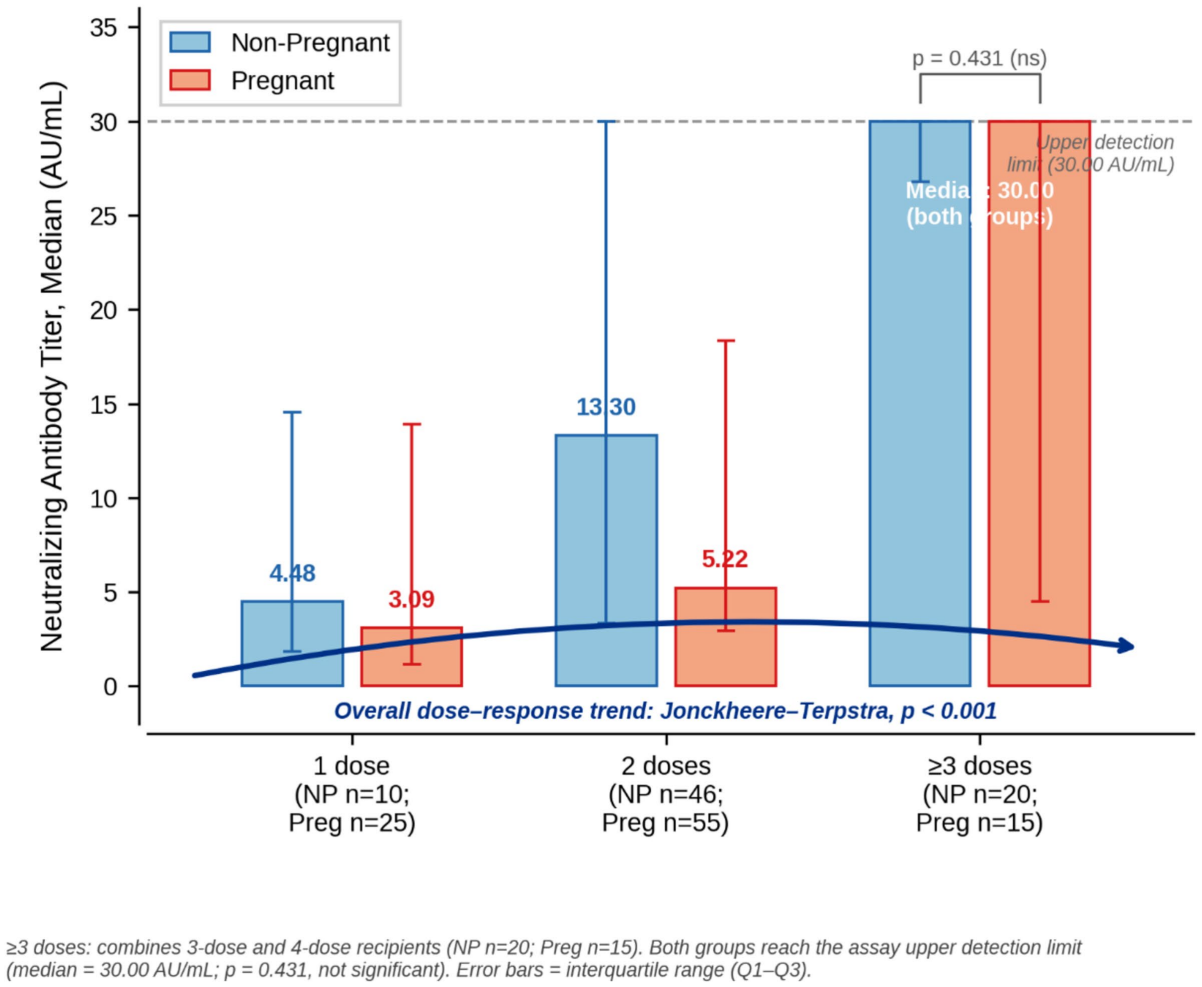
Median neutralizing antibody titers by vaccine dose category and pregnancy status. Grouped bar chart. Bars represent median values derived from the sVNT assay (AU/mL). Exact median values for each category: 1 dose—non-pregnant (NP) 4.48, pregnant (Preg) 3.09; 2 doses—NP 13.30, Preg 5.22; ≥3 doses—NP 30.00, Preg 30.00. These values correspond directly to the data presented in Table 2 (individual dose strata) and the combined ≥3-dose row. The ≥3 doses category combines the 3-dose stratum and the ≥4-dose stratum (NP: *n* = 11 + 9 = 20; Preg: *n* = 12 + 3 = 15); both groups achieve the assay ceiling (median 30.00 AU/mL), and the difference is not statistically significant (*p* = 0.431). The dashed horizontal line represents the assay upper detection limit (30.00 AU/mL). The blue arrow indicates the overall dose–response trend (Jonckheere–Terpstra test, *p* < 0.001). A stepwise increase in median antibody titers with increasing vaccine doses is evident in both groups.

Participant age was not associated with neutralizing antibody titers in the overall cohort (Spearman ρ = −0.051, *p* = 0.507) or within the pregnant and non-pregnant subgroups. This indicates that the younger age of pregnant participants does not account for their lower antibody levels. Similarly, the interval from the last vaccine dose to sampling showed only a weak and non-significant inverse correlation with antibody levels (*ρ* = −0.15, *p* = 0.06). Importantly, the actual time since the last vaccine dose did not differ significantly between non-pregnant (median 7.5 weeks, IQR 5.2–9.8) and pregnant participants (median 8.1 weeks, IQR 6.0–10.2; Mann–Whitney *U* test, *p* = 0.345), confirming that differential waning is unlikely to have biased the group comparisons (Table 1).

Among pregnant participants, gestational age at blood draw ranged from 4 to 42 weeks. Gestational age at sampling was not associated with neutralizing antibody titers (Spearman *ρ* = −0.036, *p* = 0.727) (Table 3). Consistently, antibody levels did not differ by trimester of vaccination (first, second, or third), with median titers of 5.62, 5.32, and 8.08 AU/mL, respectively (Kruskal–Wallis *H* = 0.006, *p* = 0.997). This suggests that the timing of vaccination during pregnancy was not a major determinant of the peak post-vaccination humoral response.

**Table 3:** Spearman rank correlation coefficients between antibody titers and key continuous variables.

Variable	Overall (*N* = 171)	Non-pregnant (*n* = 76)	Pregnant (*n* = 95)
Age	*ρ* = −0.051 (*p* = 0.507)	*ρ* = −0.171 (*p* = 0.140)	*ρ* = 0.007 (*p* = 0.950)
Vaccine dose count	*ρ* = 0.341 (*p* < 0.001)	*ρ* = 0.306 (*p* = 0.007)	*ρ* = 0.310 (*p* = 0.002)
Gestational age (weeks)	NA	NA	*ρ* = −0.036 (*p* = 0.727)

**Figure 3:**
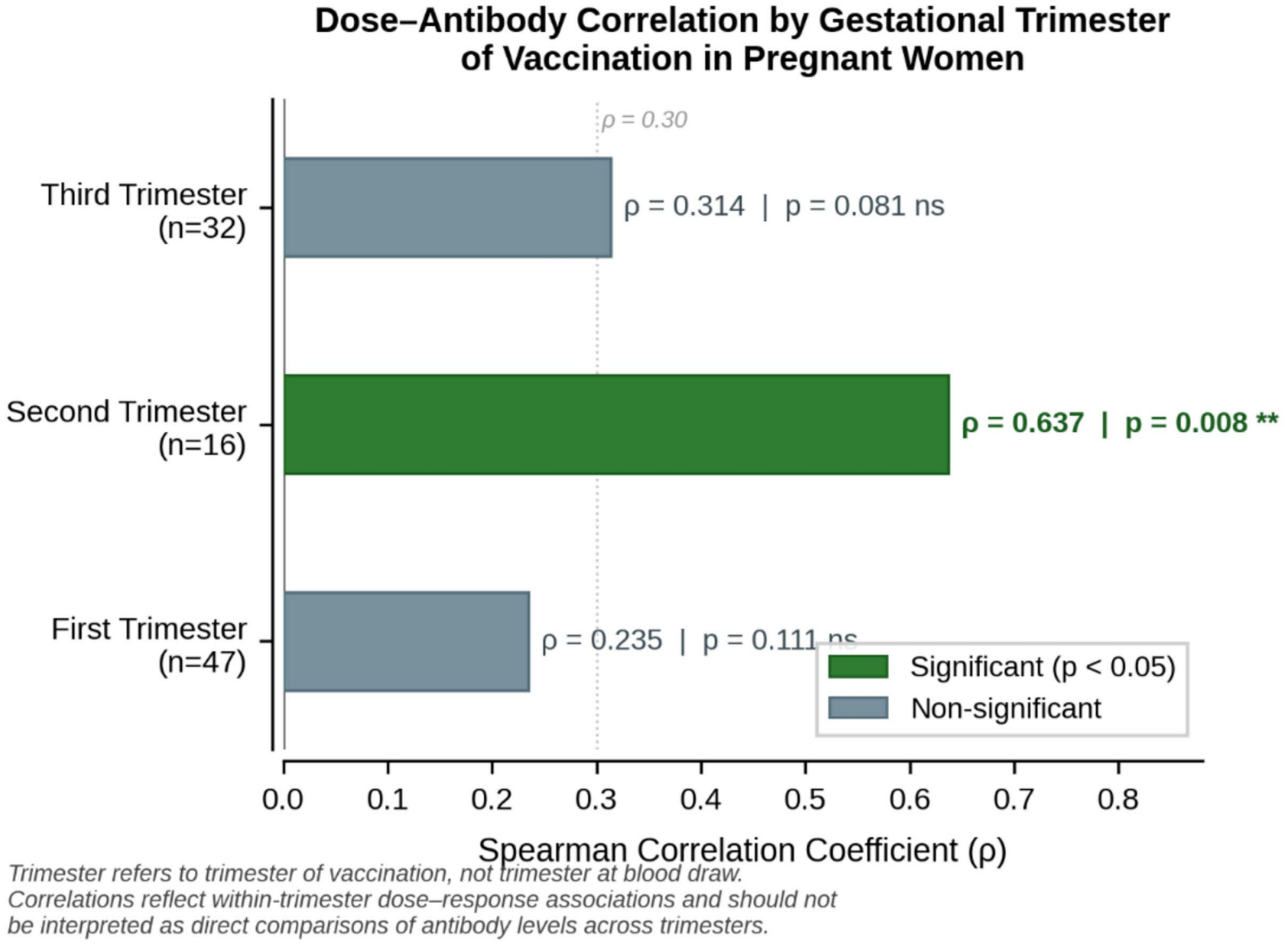
Spearman correlation between vaccine dose number and neutralizing antibody titers, stratified by trimester of vaccination (not trimester at blood draw). Horizontal bar chart. Each bar represents the Spearman correlation coefficient (*ρ*) between vaccine dose count and antibody titer within each trimester-of-vaccination subgroup of pregnant women. Green bars indicate statistically significant correlations (*p* < 0.05); gray bars represent non-significant associations. Exact values: first trimester (*n* = 47): *ρ* = 0.235, *p* = 0.111 (ns); second trimester (*n* = 16): *ρ* = 0.637, *p* = 0.008 (**); third trimester (*n* = 32): *ρ* = 0.314, *p* = 0.081 (ns). The second trimester demonstrated the strongest dose–antibody correlation, suggesting that additional doses during mid-pregnancy may be associated with a greater incremental antibody benefit. These analyses reflect within-trimester dose–response associations and should not be interpreted as direct comparisons of antibody levels across trimesters.

We next assessed whether the association between vaccine dose count and antibody level varied by the trimester of vaccination. A strong, statistically significant positive correlation was observed among women vaccinated in the second trimester (Spearman *ρ* = 0.637, *p* = 0.008), indicating substantially higher titers in those receiving more doses during mid-pregnancy. In contrast, the correlation was weaker and non-significant in first-trimester recipients (*ρ* = 0.235, *p* = 0.111) and borderline in the third trimester (*ρ* = 0.314, *p* = 0.081) (Figure 3). These differences should be interpreted cautiously given the small subgroup sizes (1st trimester: *n* = 47; 2nd trimester: *n* = 16; 3rd trimester: *n* = 32). Collectively, the findings suggest that booster dosing during pregnancy, especially in mid-gestation, may enhance antibody responses.

To evaluate independent predictors of neutralizing antibody response, we performed two multivariable regression analyses (Table 4). VIF values for all predictors ranged from 1.03 to 1.09, confirming the absence of meaningful multicollinearity. In multivariable linear regression, vaccine dose count was the only statistically significant independent predictor of log-transformed antibody titers (*β* = 0.535, 95% CI 0.242–0.829, *p* < 0.001). mRNA vaccine platform was also independently associated with higher titers (*β* = 0.721, 95% CI 0.070–1.371, *p* = 0.030). After adjustment, pregnancy status was no longer a statistically significant predictor (*β* = −0.403, 95% CI − 0.826 to 0.019, *p* = 0.061), although a non-significant trend remained. Age (*p* = 0.296) and prior SARS-CoV-2 infection (*p* = 0.981) were not independently associated with antibody levels. In the multivariable logistic regression, vaccine dose count was the dominant predictor of ceiling achievement (OR = 4.15, 95% CI 2.13–8.07, *p* < 0.001). Pregnancy status retained independent significance in this model (OR = 0.31, 95% CI 0.14–0.68, *p* = 0.003), though its effect magnitude was substantially attenuated. The differing results between the linear and logistic models reflect distinct measurement perspectives: the linear model captures continuous antibody magnitude, whereas the logistic model captures probability of ceiling attainment, which is disproportionately influenced by cumulative vaccine exposure. Collectively, these analyses confirm that differences in cumulative vaccine exposure are the primary determinant of antibody response magnitude, and that lower antibody levels in pregnant women are driven predominantly by reduced exposure, although a residual independent association of pregnancy remains possible.

**Table 4:** Multivariable regression analyses: predictors of neutralizing antibody response.

Panel A: multivariable linear regression (dependent variable: log-transformed antibody titer).				
Variable	*β* coeff.	95% CI	*p*-value	VIF
Pregnancy status	−0.403	−0.826 to 0.019	0.061	1.09
Vaccine dose count	0.535	0.242 to 0.829	<0.001	1.08
Age (years)	−0.019	−0.054 to 0.017	0.296	1.05
Prior SARS-CoV-2 infection	0.006	−0.454 to 0.465	0.981	1.05
mRNA vaccine platform	0.721	0.070 to 1.371	0.030	1.03

Panel B: multivariable logistic regression (dependent variable: reaching assay upper detection limit ≥30 AU/mL)			
Variable	OR	95% CI	*p*-value
Pregnancy status	0.31	0.14–0.68	0.003
Vaccine dose count	4.15	2.13–8.07	<0.001
Age (years)	0.97	0.91–1.04	0.363
Prior SARS-CoV-2 infection	0.86	0.36–2.06	0.733
mRNA vaccine platform	3.76	0.76–18.62	0.105

*R*^2^ = 0.123, adj. *R*^2^ = 0.095, *F* (5,157) = 4.39, *p* = 0.001. All VIF values <2—no meaningful multicollinearity.

Pseudo *R*^2^ = 0.193, LLR *p* < 0.001. Ceiling achievement (≥30 AU/mL): 43.4% NP vs. 18.9% Preg. Wide CI for mRNA OR reflects small inactivated-vaccine subgroup (*n* = 18). *β*, unstandardized regression coefficient; CI, confidence interval; OR, odds ratio; VIF, variance inflation factor. Models on *n* = 163 (eight heterologous excluded).

To assess the potential confounding effect of prior SARS-CoV-2 infection, we performed a sensitivity analysis restricted to participants with no confirmed or suspected prior SARS-CoV-2 infection (*n* = 110: 46 non-pregnant and 64 pregnant; 14 participants with uncertain infection history were additionally excluded). The primary finding was fully preserved: pregnant women had significantly lower neutralizing antibody titers than non-pregnant women (median 5.42 vs. 25.00 AU/mL; Mann–Whitney *U* test, *p* = 0.008) and received fewer vaccine doses on average (mean doses: pregnant 1.89 vs. non-pregnant 2.46; *p* = 0.001). The higher median titer among infection-naive non-pregnant participants (25.00 AU/mL) relative to the full non-pregnant cohort (16.25 AU/mL) reflects the selective exclusion of participants with prior infection, who had comparatively lower titers (median 13.20 AU/mL), thereby redistributing the subgroup median upward. Within each group, antibody titers did not differ significantly between participants with and without prior infection (non-pregnant: 13.20 vs. 25.00 AU/mL, *p* = 0.389; pregnant: 5.75 vs. 5.42 AU/mL, *p* = 0.669), confirming that adjustment for documented prior infection did not materially alter the primary findings.

After excluding 8 individuals who received mixed-platform (heterologous) regimens, median antibody titers were numerically higher among the 145 mRNA vaccine recipients compared to the 18 inactivated vaccine recipients (11.30 vs. 7.72 AU/mL; *p* = 0.165). The heterologous group, which had the highest median dose count (4 doses), also had the highest median antibody titers (13.81 AU/mL), suggesting higher antibody levels were driven by cumulative dose number rather than platform synergy.

No severe COVID-19 cases occurred during follow-up. Seven mild breakthrough infections were documented (5 pregnant, 2 non-pregnant), with no significant difference in rates between groups (5.3% vs. 2.6%, *p* = 0.68). Pregnancy and neonatal outcomes for 92 deliveries were consistent with institutional norms, and no serious vaccine-related adverse events were reported.

## Discussion

In this cohort study comparing the immunogenicity of COVID-19 vaccination in pregnant and non-pregnant women, we observed significantly lower unadjusted neutralizing antibody levels among pregnant participants. However, our analyses indicate that this difference is unlikely to be primarily driven by intrinsic pregnancy-related immune impairment and is more plausibly explained by differences in cumulative vaccine exposure. The positive association between neutralizing antibody titers and vaccine dose count was robust in the overall cohort and remained significant when analyses were performed separately in both subgroups. Although a modest residual independent contribution of pregnancy, as suggested by the logistic regression findings, cannot be entirely excluded, these findings support the conclusion that overall humoral responsiveness remains preserved during pregnancy despite quantitative differences in antibody magnitude and that, when vaccine exposure is comparable, pregnant and non-pregnant women achieve similar antibody levels. It is important to acknowledge that these conclusions are based on neutralizing antibody titers and do not encompass the full spectrum of SARS-CoV-2–specific immunity.

To place these observations in appropriate biological and clinical context, it is essential to consider the broader literature on the immunogenicity of COVID-19 vaccination during pregnancy.

### Immunogenicity of COVID-19 vaccination in pregnancy in the context of existing literature

Pregnancy is characterized by a complex immunological adaptation rather than a state of global immune suppression (12). This adaptation involves finely regulated shifts in both innate and adaptive immune function, including modulation of T-helper cell polarization, expansion of regulatory T-cell populations, and functional changes in antigen-presenting cells, all of which facilitate fetomaternal tolerance while preserving effective host defense mechanisms (8, 12). Early in the COVID-19 pandemic, concerns were raised that these physiological immune adaptations might attenuate vaccine-induced immunity in pregnant individuals. However, accumulating evidence from prospective cohort studies has largely alleviated these concerns (1, 2).

Seminal investigations have demonstrated that pregnant individuals mount robust humoral and cellular immune responses following mRNA COVID-19 vaccination. Gray et al. (1) first showed that mRNA vaccination during pregnancy elicits strong neutralizing antibody responses effectively transmitted across the placenta. Collier et al. (2) reported preserved neutralizing antibody and T-cell responses following a two-dose mRNA vaccine series. Our findings extend this body of literature by providing real-world evidence that lower population-level antibody titers in pregnant women reflect differential vaccine exposure rather than impaired immune capacity.

### Cumulative vaccine exposure as the primary determinant of antibody response

One of the most consistent findings of our study is the strong positive association between neutralizing antibody titers and the total number of COVID-19 vaccine doses received. These findings are highly concordant with prior studies demonstrating that cumulative antigen exposure through booster vaccination substantially enhances neutralizing antibody responses, including against immune-evasive variants of concern (3, 15, 16). Atyeo et al. (6) showed that administration of a booster dose during pregnancy markedly increased neutralizing antibody titers against the Omicron variant, achieving levels comparable to those in non-pregnant individuals. Participants in our cohort who received three or more vaccine doses exhibited the highest antibody levels, frequently reaching the upper limit of assay detection, irrespective of pregnancy status.

Interpretation of antibody magnitude at higher dose categories is influenced by the upper detection limit of the assay, resulting in right-censoring at values ≥30 AU/mL. This ceiling effect may attenuate the apparent differences between groups at higher dose levels and should be considered when interpreting the logistic regression results, particularly the wide confidence interval for the mRNA platform odds ratio (OR 3.76, 95% CI 0.76–18.62), which reflects the small inactivated-vaccine subgroup (*n* = 18).

Critically, when antibody responses were stratified by dose category, the differences between pregnant and non-pregnant women were no longer statistically significant. This finding suggests that intrinsic pregnancy-related immune impairment is unlikely to be the primary driver of the observed group differences. It is important to note that the categorical distribution of dose counts was broadly similar between groups (*χ*^2^ *p* = 0.098); it is the continuous measure of cumulative vaccine exposure and the ordinal trend toward fewer high-dose recipients in the pregnant cohort (linear-by-linear *p* = 0.004) that more sensitively captures the biological dose–response relationship. Although categorical dose distributions were similar, cumulative exposure differed when assessed as a continuous variable, which better reflects the biological dose–response gradient underlying the observed antibody differences. This interpretation is supported by epidemiologic data demonstrating persistently lower uptake of additional vaccine doses among pregnant individuals, frequently attributable to safety concerns, risk perception, and inadequate counseling (13, 17).

In addition, although the interval between the last vaccine dose and antibody sampling showed a weak inverse association with antibody levels, the actual sampling interval did not differ significantly between pregnant and non-pregnant participants, suggesting that differential antibody waning is unlikely to be a major driver of the observed group-level differences, although subgroup-level contributions cannot be fully excluded (see Limitations).

### Timing of vaccination during pregnancy and trimester-specific effects

Beyond overall vaccine exposure, the timing of vaccination during pregnancy represents another dimension with potential immunologic relevance. In our study, gestational age at vaccination did not significantly influence peak neutralizing antibody levels, and no meaningful differences were observed across trimesters (*p* = 0.997). This finding is reassuring and is consistent with prior reports (1, 2, 14, 16, 17).

Notably, the magnitude of the dose–antibody correlation varied by trimester of vaccination. A strong and statistically significant correlation was observed in second-trimester recipients (*ρ* = 0.637, *p* = 0.008), whereas this association was weaker in the first trimester (*ρ* = 0.235, *p* = 0.111) and borderline in the third trimester (*ρ* = 0.314, *p* = 0.081). Although constrained by limited subgroup sizes, these findings raise the hypothesis that mid-gestation may represent a period of heightened responsiveness to additional vaccine doses. This observation warrants confirmation in larger, prospective studies and should be interpreted as hypothesis-generating rather than definitive.

### Vaccine platform and antibody responses

We next explored whether vaccine platform contributed to observed differences in immunogenicity. In unadjusted comparisons, mRNA vaccines were associated with numerically higher median antibody titers than inactivated vaccines (11.30 vs. 7.72 AU/mL; *p* = 0.165). In multivariable analysis, mRNA platform was independently associated with higher titers (*β* = 0.721, *p* = 0.030). However, platform-related effects should be interpreted cautiously given the limited statistical power arising from the small inactivated-vaccine subgroup (*n* = 18), as reflected by the wide confidence interval of the mRNA platform odds ratio in the logistic model (OR 3.76, 95% CI 0.76–18.62). The heterologous group, which also had the greatest cumulative dose exposure (median 4 doses), demonstrated the highest median antibody titers (13.81 AU/mL), suggesting that total dose number rather than platform synergy most likely accounted for the elevated levels (2, 12, 13). Our findings indicate that, in real-world settings, ensuring completion of all recommended vaccine doses may be more critical for achieving optimal antibody-mediated immunity than vaccine platform selection alone.

### Clinical and public health implications

Our data demonstrate that pregnant women are fully capable of mounting strong neutralizing antibody responses following COVID-19 vaccination. The lower antibody levels observed among pregnant participants reflect modifiable differences in cumulative vaccine exposure rather than biologically constrained immune responsiveness. Accordingly, targeted interventions aimed at improving uptake of additional vaccine doses among pregnant individuals represent a highly effective strategy for eliminating observed gaps in population-level immunity.

In addition, maternal antibody concentrations have direct implications for neonatal protection through transplacental transfer of SARS-CoV-2-specific IgG antibodies. By promoting completion of recommended vaccine schedules and timely additional dose administration during pregnancy, clinicians can meaningfully enhance protection for both the mother and the infant. It is important to acknowledge that our findings are limited to neutralizing antibody responses and do not capture the full spectrum of SARS-CoV-2-specific humoral or cellular immunity. T-cell responses and memory B-cell populations, which are critical for durable protection, were not assessed in this study. Accordingly, the conclusions regarding preserved immune responsiveness during pregnancy should be understood in this specific context.

No severe COVID-19 cases were observed during follow-up, and the incidence of mild breakthrough infections did not differ significantly between pregnant and non-pregnant participants. Although this study was not powered to assess clinical vaccine effectiveness, these observations provide reassuring contextual evidence.

### Study limitations

Several limitations should be acknowledged. First, as an observational study, residual confounding cannot be entirely excluded. Second, prior SARS-CoV-2 infection was ascertained by self-report and clinical records; anti-nucleocapsid (anti-N) serological testing was not performed. Accordingly, unrecognized asymptomatic infections may have been misclassified. However, the balanced prior infection rates between groups (*p* = 0.256) and the preserved sensitivity analysis findings argue against material bias. Third, although blood samples were collected within a predefined uniform 4–12 week window for all participants (with no significant difference in median sampling time between the overall pregnant and non-pregnant cohorts), stratified analyses of sampling interval balance within individual dose categories were not performed, and differential waning within dose strata cannot be formally excluded. The weak and non-significant inverse correlation (*ρ* = −0.15, *p* = 0.06) argues against a major overall contribution of differential waning. Fourth, antibody titers were reported in AU/mL without conversion to WHO International Units, limiting comparability with studies using the NIBSC 20/136 standard. Fifth, subgroup analyses stratified by trimester were constrained by limited sample sizes. Sixth, our study focused on neutralizing antibody responses and did not assess cellular immune responses, which are critical for long-term protection. Seventh, the wide confidence interval for the mRNA platform OR in the logistic model (0.76–18.62) reflects the small inactivated-vaccine subgroup (*n* = 18) and precludes firm platform-specific conclusions. Eighth, higher serial dilutions were not performed, limiting the assay’s dynamic range and resulting in right-censoring at ≥30 AU/mL; this was addressed analytically through logistic regression of ceiling achievement.

While multivariable analyses attenuated the association between pregnancy status and antibody magnitude, the presence of a residual effect in the logistic model suggests that both differences in cumulative vaccine exposure and potential pregnancy-related biological factors may contribute to the observed differences. Future studies should incorporate longitudinal designs with larger sample sizes, evaluate both humoral and cellular immunity, and examine the durability of vaccine-induced protection across pregnancy and the postpartum period.

## Conclusion

This study demonstrates that COVID-19 vaccination elicits strong neutralizing antibody responses in pregnant women, with overall humoral responsiveness preserved despite quantitative differences in antibody magnitude. The lower antibody levels observed in the pregnant cohort are largely attributable to differences in cumulative vaccine exposure rather than pregnancy-associated immune modulation, although a residual independent effect of pregnancy cannot be definitively excluded. These conclusions are based on neutralizing antibody titers and do not encompass the full spectrum of vaccine-induced immunity. When vaccine exposure is equivalent, pregnant and non-pregnant women achieve comparable antibody levels. These findings, together with extensive evidence supporting vaccine safety in pregnancy, underscore the importance of ensuring that pregnant individuals receive all recommended COVID-19 vaccine doses to maximize protection for both mother and infant.

## Data availability statement

The raw data supporting the conclusions of this article will be made available by the authors, without undue reservation.
